# Supplementary material for: The nuclear factor ID3 endows macrophages with a potent anti-tumour activity
Source: Nature. 2024 Feb 7;626(8000):864–73. doi: 10.1038/s41586-023-06950-4 (PMC10881399; doi:10.1038/s41586-023-06950-4)
Supplement: Supplementary file 1 — Supplementary Figs. 1–3 and Supplementary Tables 1 and 2. [file 41586_2023_6950_MOESM1_ESM.pdf]

nature portfolio

https://doi.org/10.1038/s41586-023-06950-4

Supplementary information

The nuclear factor ID3 endows macrophages with a potent anti-tumour activity

In the format provided by the
authors and unedited

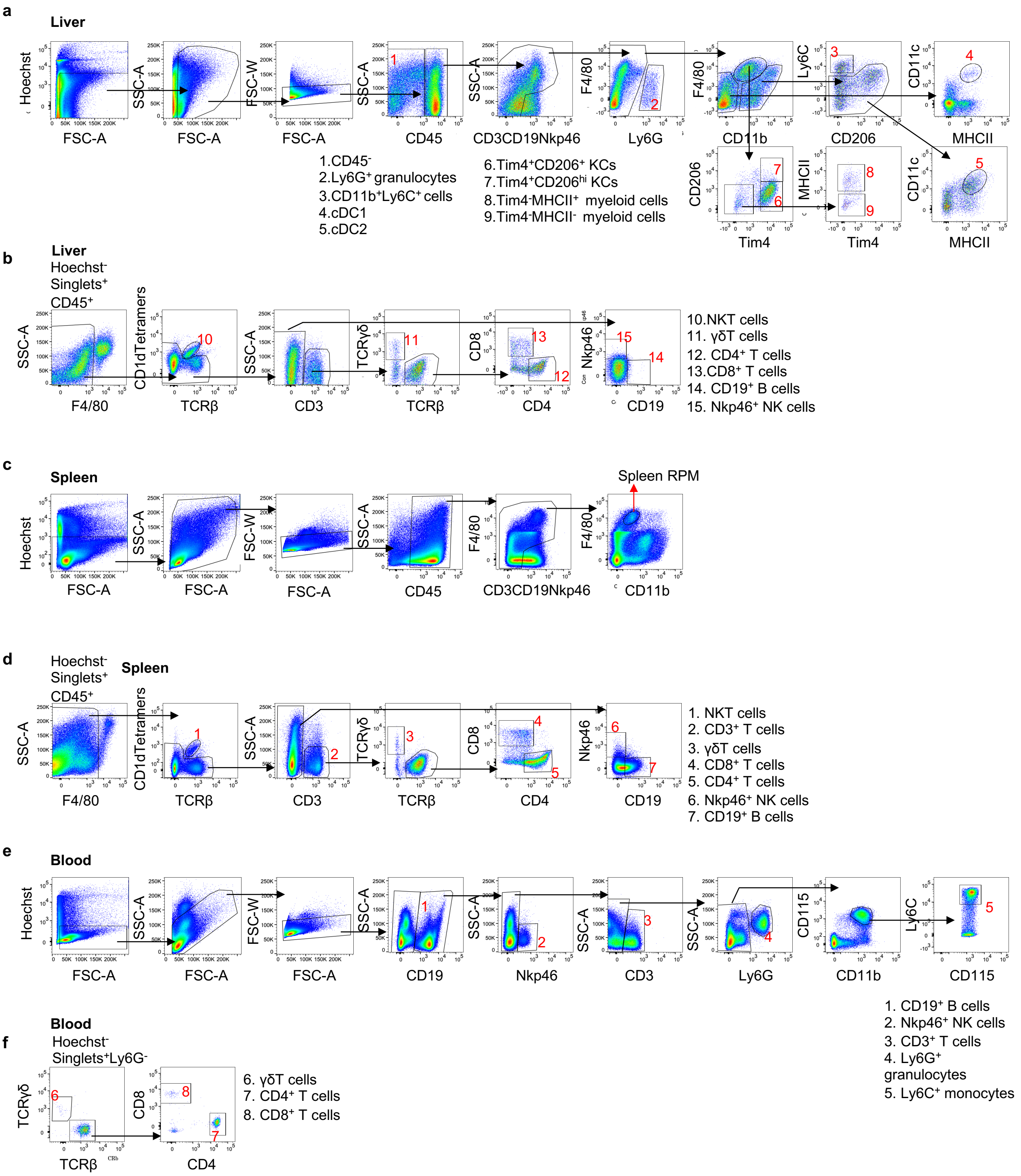

Supplementary information figure 1. Flow cytometry gating strategies for immune cells in liver, spleen, and blood. **a,b-** Representative gating strategies for liver CD45^{-} cells, Ly6G^{+} granulocytes , CD11b^{+}Ly6C^{+} cells, cDC1, cDC2, Tim4^{+}CD206^{+} KCs, Tim4^{+}CD206^{hi} KCs, Tim4^{-}MHCII^{+} myeloid cells, Tim4^{-}MHCII^{-} myeloid cells , NKT cells, γδT cells, CD4^{+} T cells, CD8^{+} T cells , CD19^{+} B cells , Nkp46^{+} NK cells. **c,d-** Representative gating strategies for spleen red pulp macrophages (RPM), NKT cells , CD3^{+} T cells , γδT cells , CD8^{+} T cells , CD4^{+} T cells , Nkp46^{+} NK cells , CD19^{+} B cells. **e,f-** Representative gating strategies for blood , CD19^{+} B cells , Nkp46^{+} NK cells , CD3^{+} T cells , Ly6G^{+} granulocytes , Ly6C^{+} monocytes , γδT cells , CD4^{+} T cells , CD8^{+} T cells. Related to Figure 2b, 3c, 3d, 3e, 4i, 4k, 5g, 5i, 5j, 6h. Extended data Figure 1a, 1c, 1d, 1e, 1f, 1h, 1i, 2h, 2l, 2k, 2l, 2o, 5c, 5d, 6a, 6e, 6f, 6g, 7e, 7j, 10c.

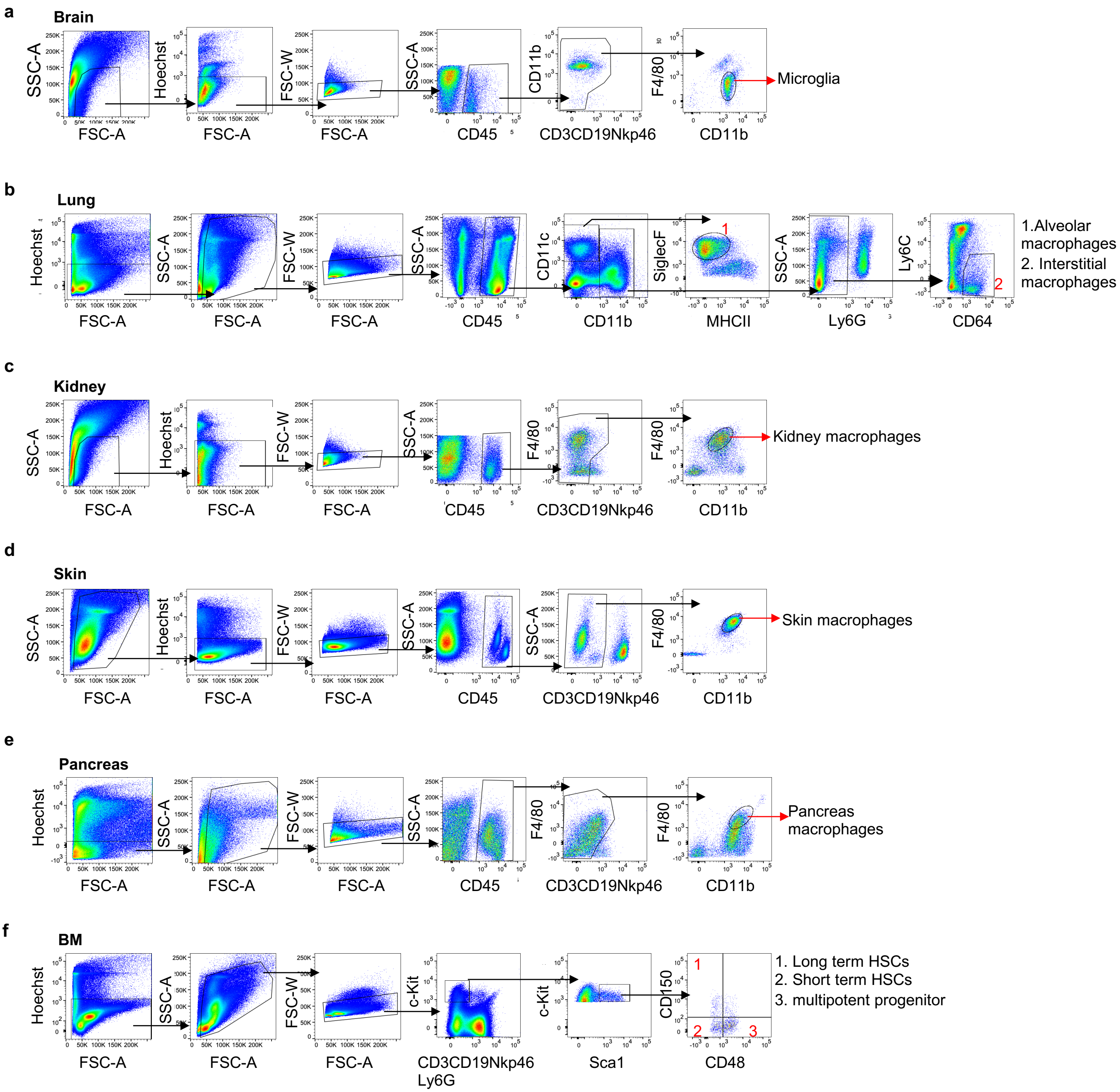

Supplementary information figure 2. Flow cytometry gating strategies for macrophages in brain, lung, kidney, skin, pancreas, and hematopoietic stem cells in bone marrow. a- Representative gating strategies for brain microglia cells. **b-** Representative gating strategies for Alveolar macrophages, Interstitial macrophages. **c-** Representative gating strategies for kidney macrophages. **d-** Representative gating strategies for skin macrophages. **e-** Representative gating strategies for pancreas macrophages. **f-** Representative gating strategies for bone marrow Long term hematopoietic stem cells(LT-HSCs), Short term hematopoietic stem cells(ST-HSCs), multipotent progenitor. Related to Extended data figure 1f, 2o.

.

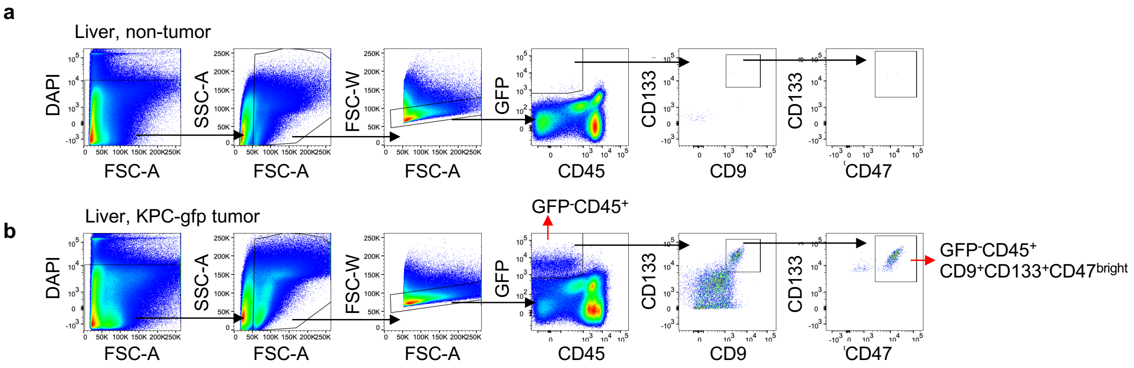

Supplementary information figure 3. Flow cytometry gating strategies for GFP$^-$CD45$^+$ tumor cells and GFP$^-$CD45$^+$CD9$^+$CD133$^+$CD47^{bright} metastatic initiating tumor cells in tumor bearing liver.

a,b- Representative gating strategies for GFP$^-$CD45$^+$ tumor cells and GFP$^-$CD45$^+$CD9$^+$CD133$^+$CD47^{bright} metastatic initiating tumor cells in liver from non-tumor bearing mice (**a**) or tumor bearing mice 2 weeks after intra-portal injection of KPC-gfp cells (**b**). Related to Figure 1h, 3i, 4f. Extended data Figure 1n, 5f.

Figures	Y title	Group1	Group2	Group2 vs Group1 p Value.
Figure 1c	Photo Radiance	*Csf1r*$^{f/f}$_*liver*	*Clec4f*^{Cre} *Csf1r*$^{f/f}$_*liver*	2.9E-05
Figure 1e	Photo Radiance	*R26*$^{LSL\text{-}DTR}$_*liver*	*Clec4f*^{Cre} *R26*$^{LSL\text{-}DTR}$_*liver*	1.3E-06
Figure 1e	Photo Radiance	*R26*$^{LSL\text{-}DTR}$_*lung*	*Clec4f*^{Cre} *R26*$^{LSL\text{-}DTR}$_*lung*	2.0E-06
Figure 2b	% Partner derived (45.1^{+})	Tim4^{+}_No tumor	Tim4^{-} MHCII^{-}_No tumor	8.4E-06
Figure 2d	% Tim4^{+} tdT^{+}	>500μm	0-50μm	9.7E-06
Figure 2e	tdT relative MFI	Lamp1^{+} lysosome	Lamp1^{-} region	<1E-15
Figure 2h	CCL3_Relative MFI	*>50μm*	*0-50μm*	<1E-15
	CCL3_Relative MFI	*>50μm*	core	<1E-15
	CCL4_Relative MFI	*>50μm*	*0-50μm*	<1E-15
	CCL4_Relative MFI	*>50μm*	core	<1E-15
	CCL5_Relative MFI	*>50μm*	*0-50μm*	<1E-15
	CCL5_Relative MFI	*>50μm*	core	<1E-15
	CCL5_Relative MFI	*0-50μm*	core	2.5E-09
	IL12P70_Relative MFI	*>50μm*	*0-50μm*	<1E-15
	IL12P70_Relative MFI	*>50μm*	core	<1E-15
	IL12P70_Relative MFI	*0-50μm*	core	<1E-15
	IL15_Relative MFI	*>50μm*	*0-50μm*	<1E-15
	IL15_Relative MFI	*>50μm*	core	<1E-15
	IL15_Relative MFI	*0-50μm*	core	<1E-15
	IL18_Relative MFI	*>50μm*	*0-50μm*	<1E-15
	IL18_Relative MFI	*>50μm*	core	<1E-15
	IL18_Relative MFI	*0-50μm*	core	<1E-15
Figure 2i	CD8^{+} T cells. *mm*$^{-2}$ (x10^{3})	*>50μm*	*0-50μm*	2.2E-06
	CD8^{+} T cells. *mm*$^{-2}$ (x10^{3})	*0-50μm*	core	1.7E-05
Figure 2j	Nkp46^{+} Lamp1^{+}. mm-2 (x10^{3})	*>50μm*	*0-50μm*	8.2E-05
Figure 4d	SIRPA relative MFI	*Id3*$^{f/f}$_*tumor*	*Clec4f*^{Cre} *Id3*$^{f/f}$_*tumor*	4.9E-05
	DECTIN1 relative MFI	*Id3*$^{f/f}$_*tumor*	*Clec4f*^{Cre} *Id3*$^{f/f}$_*tumor*	2.1E-05
Figure 4e	% Tim4^{+} GFP^{+}	*Id3*$^{f/f}$_*>500μm*	*Id3*$^{f/f}$_*0-50μm*	1.0E-10
	% Tim4^{+} GFP^{+}	*Id3*$^{f/f}$_*50-500μm*	*Id3*$^{f/f}$_*0-50μm*	5.4E-09
	% Tim4^{+} GFP^{+}	*Id3*$^{f/f}$_*50-500μm*	*Clec4f*^{Cre} *Id3*$^{f/f}$_*50-500μm*	1.2E-05
Figure 4f	%Tim4^{+} GFP^{+}	*Id3*$^{f/f}$	*Clec4f*^{Cre} *Id3*$^{f/f}$	5.0E-05
Figure 4h	CCL3 relative MFI	*Id3*$^{f/f}$_*>50μm*	*Id3*$^{f/f}$_*0-50μm*	<1E-15
	CCL3 relative MFI	*Id3*$^{f/f}$_*>50μm*	*Id3*$^{f/f}$_*core*	<1E-15
	CCL3 relative MFI	*Id3*$^{f/f}$_*0-50μm*	*Clec4f*^{Cre} *Id3*$^{f/f}$_*0-50μm*	<1E-15
	CCL3 relative MFI	*Id3*$^{f/f}$_*core*	*Clec4f*^{Cre} *Id3*$^{f/f}$_*core*	<1E-15
	CCL4 relative MFI	*Id3*$^{f/f}$_*>50μm*	*Id3*$^{f/f}$_*0-50μm*	<1E-15
	CCL4 relative MFI	*Id3*$^{f/f}$_*>50μm*	*Id3*$^{f/f}$_*core*	<1E-15
	CCL4 relative MFI	*Id3*$^{f/f}$_*0-50μm*	*Clec4f*^{Cre} *Id3*$^{f/f}$_*0-50μm*	<1E-15
	CCL4 relative MFI	*Id3*$^{f/f}$_*core*	*Clec4f*^{Cre} *Id3*$^{f/f}$_*core*	<1E-15
	CCL5 relative MFI	*Id3*$^{f/f}$_*>50μm*	*Id3*$^{f/f}$_*0-50μm*	<1E-15
	CCL5 relative MFI	*Id3*$^{f/f}$_*>50μm*	*Id3*$^{f/f}$_*core*	<1E-15
	CCL5 relative MFI	*Id3*$^{f/f}$_*0-50μm*	*Clec4f*^{Cre} *Id3*$^{f/f}$_*0-50μm*	<1E-15
	CCL5 relative MFI	*Id3*$^{f/f}$_*core*	*Clec4f*^{Cre} *Id3*$^{f/f}$_*core*	<1E-15
	IL12 relative MFI	*Id3*$^{f/f}$_*>50μm*	*Id3*$^{f/f}$_*0-50μm*	<1E-15
	IL12 relative MFI	*Id3*$^{f/f}$_*>50μm*	*Id3*$^{f/f}$_*core*	<1E-15
	IL12 relative MFI	*Id3*$^{f/f}$_*0-50μm*	*Clec4f*^{Cre} *Id3*$^{f/f}$_*0-50μm*	<1E-15
	IL12 relative MFI	*Id3*$^{f/f}$_*core*	*Clec4f*^{Cre} *Id3*$^{f/f}$_*core*	<1E-15
	IL15 relative MFI	*Id3*$^{f/f}$_*>50μm*	*Id3*$^{f/f}$_*0-50μm*	<1E-15
	IL15 relative MFI	*Id3*$^{f/f}$_*>50μm*	*Id3*$^{f/f}$_*core*	<1E-15
	IL15 relative MFI	*Id3*$^{f/f}$_*0-50μm*	*Clec4f*^{Cre} *Id3*$^{f/f}$_*0-50μm*	<1E-15
	IL15 relative MFI	*Id3*$^{f/f}$_*core*	*Clec4f*^{Cre} *Id3*$^{f/f}$_*core*	<1E-15
	IL18 relative MFI	*Id3*$^{f/f}$_*>50μm*	*Id3*$^{f/f}$_*0-50μm*	<1E-15
	IL18 relative MFI	*Id3*$^{f/f}$_*>50μm*	*Id3*$^{f/f}$_*core*	<1E-15
	IL18 relative MFI	*Id3*$^{f/f}$_*0-50μm*	*Clec4f*^{Cre} *Id3*$^{f/f}$_*0-50μm*	<1E-15
	IL18 relative MFI	*Id3*$^{f/f}$_*core*	*Clec4f*^{Cre} *Id3*$^{f/f}$_*core*	<1E-15
Figure 4i	CD8+ cell / g (x10^{5})	*Id3*$^{f/f}$	*Clec4f*^{Cre} *Id3*$^{f/f}$	3.5E-06
Figure 4j	Nkp46^{+} Lamp1^{+} cells *mm*$^{-2}$ (x10^{3})	*Id3*$^{f/f}$_*>50μm*	*Id3*$^{f/f}$_*0-50μm*	2.8E-11
	Nkp46^{+} Lamp1^{+} cells *mm*$^{-2}$ (x10^{3})	*Id3*$^{f/f}$_*>50μm*	*Id3*$^{f/f}$_*core*	4.4E-08
	Nkp46^{+} Lamp1^{+} cells *mm*$^{-2}$ (x10^{3})	*Id3*$^{f/f}$_*0-50μm*	*Clec4f*^{Cre} *Id3*$^{f/f}$_*0-50μm*	3.2E-09
	Nkp46^{+} Lamp1^{+} cells *mm*$^{-2}$ (x10^{3})	*Id3*$^{f/f}$_*core*	*Clec4f*^{Cre} *Id3*$^{f/f}$_*core*	2.3E-06
	CD8^{+} T cells *mm*$^{-2}$ (x10^{3})	*Id3*$^{f/f}$_*>50μm*	*Id3*$^{f/f}$_*0-50μm*	<1E-15
	CD8^{+} T cells *mm*$^{-2}$ (x10^{3})	*Id3*$^{f/f}$_*>50μm*	*Id3*$^{f/f}$_*core*	4.9E-10
	CD8^{+} T cells *mm*$^{-2}$ (x10^{3})	*Id3*$^{f/f}$_*0-50μm*	*Clec4f*^{Cre} *Id3*$^{f/f}$_*0-50μm*	<1E-15
	CD8^{+} T cells *mm*$^{-2}$ (x10^{3})	*Id3*$^{f/f}$_*core*	*Clec4f*^{Cre} *Id3*$^{f/f}$_*core*	1.0E-08
Figure 5f	IL12b relative mRNA	wt	wt, KPC1+ IgG	5.9E-05
Figure 5h	CD8^{+} T cells *mm*$^{-2}$ (x10^{3})	*Id3*$^{f/f}$ + IgG _*>50μm*	*Id3*$^{f/f}$ + IgG _*0-50μm*	1.8E-14
	CD8^{+} T cells *mm*$^{-2}$ (x10^{3})	*Id3*$^{f/f}$ + IgG _*>50μm*	*Id3*$^{f/f}$ + IgG _*core*	9.1E-09
	CD8^{+} T cells *mm*$^{-2}$ (x10^{3})	*Id3*$^{f/f}$ + IgG _*0-50μm*	*Id3*$^{f/f}$ + IgG _*core*	1.8E-14
	CD8^{+} T cells *mm*$^{-2}$ (x10^{3})	*Id3*$^{f/f}$ + IgG _*0-50μm*	*Clec4f*^{Cre} *Id3*$^{f/f}$ + IgG _*0-50μm*	1.8E-14
	CD8^{+} T cells *mm*$^{-2}$ (x10^{3})	*Clec4f*^{Cre} *Id3*$^{f/f}$ + IgG _ 0-50μm	*Clec4f*^{Cre} *Id3*$^{f/f}$ + α-SIRPA _*0-50μm*	1.8E-14
	CD8^{+} T cells *mm*$^{-2}$ (x10^{3})	*Clec4f*^{Cre} *Id3*$^{f/f}$ + IgG_core	*Clec4f*^{Cre} *Id3*$^{f/f}$ + α-SIRPA_*core*	1.9E-07
	Nkp46^{+}Lamp1^{+} *mm*$^{-2}$ (x10^{3})	*Id3*$^{f/f}$ + IgG_*>50μm*	*Id3*$^{f/f}$ + IgG_ *0-50μm*	7.2E-14
	Nkp46^{+}Lamp1^{+} *mm*$^{-2}$ (x10^{3})	*Id3*$^{f/f}$ + IgG_*>50μm*	*Id3*$^{f/f}$ + IgG_*core*	2.6E-07
	Nkp46^{+}Lamp1^{+} *mm*$^{-2}$ (x10^{3})	*Id3*$^{f/f}$ + IgG _*0-50μm*	*Id3*$^{f/f}$ + IgG_*core*	3.8E-08
	Nkp46^{+}Lamp1^{+} *mm*$^{-2}$ (x10^{3})	*Id3*$^{f/f}$ + IgG _*0-50μm*	*Clec4f*^{Cre} *Id3*$^{f/f}$ + IgG _*0-50μm*	2.1E-09
	Nkp46^{+}Lamp1^{+} *mm*$^{-2}$ (x10^{3})	*Clec4f*^{Cre} *Id3*$^{f/f}$ + IgG _ 0-50μm	*Clec4f*^{Cre} *Id3*$^{f/f}$ + α-SIRPA _ 0-50μm	5.2E-09
	Nkp46^{+}Lamp1^{+} *mm*$^{-2}$ (x10^{3})	*Clec4f*^{Cre} *Id3*$^{f/f}$ + IgG_core	*Clec4f*^{Cre} *Id3*$^{f/f}$ + α-SIRPA_*core*	2.0E-05
Figure 5m	SIRPA relative MFI	*Id3*$^{+/+}$_*Scramble*	*Id3*$^{-/-}$_*Scramble*	1.0E-08
	SIRPA relative MFI	*Id3*$^{+/+}$_*Scramble*	*Id3*$^{+/+}$_*sh-Elk1*	6.7E-06
	SIRPA relative MFI	*Id3*$^{+/+}$_*Scramble*	*Id3*$^{+/+}$_*sh-E2A*	8.0E-06
	SIRPA relative MFI	*Id3*$^{-/-}$_*Scramble*	*Id3*$^{-/-}$_*sh-Elk1*	4.0E-10
	SIRPA relative MFI	*Id3*$^{-/-}$_*Scramble*	*Id3*$^{-/-}$_*sh-E2A*	4.5E-09
Figure 5n	SIRPA relative MFI	Control	Lenti-Id3	8.3E-14
	SIRPA relative MFI	Scramble	sh-E2A	1.1E-13
	SIRPA relative MFI	Scramble	sh-Elk1	7.5E-14
Figure 6b	Dectin1 Relative mRNA	Lenti-Control	Lenti-hId3	4.8E-06
Figure 6c	*Ccl3* Relative mRNA	Lenti-hId3+*nt*	Lenti-hId3+*Panc1*	3.8E-08
	Ccl3 Relative mRNA	Lenti-Ctr+*Panc1*	Lenti-hId3+*Panc1*	1.8E-06
	Ccl4 Relative mRNA	Lenti-Ctr+*nt*	Lenti-Ctr+*Panc1*	2.2E-05
	Ccl4 Relative mRNA	Lenti-hId3+*nt*	Lenti-hId3+*Panc1*	2.0E-07
	IL15 Relative mRNA	Lenti-Ctr+*nt*	Lenti-Ctr+*Panc1*	4.2E-05
	IL15 Relative mRNA	Lenti-hId3+*nt*	Lenti-hId3+*Panc1*	7.1E-07
	IL18 Relative mRNA	Lenti-hId3+*nt*	Lenti-hId3+*Panc1*	2.8E-06
Figure 6d	CFSE^{-} proliferation/%	Lenti-Ctr+*Panc1*	Lenti-hId3+*Panc1*	2.8E-06
	CFSE^{-} proliferation/%	Lenti-hId3+*nt*	Lenti-hId3+*Panc1*	4.7E-07
Figure 6e	hCD8^{+} T Cell IFN-γ relative MFI	Lenti-Ctr+*Panc1*	Lenti-hId3+*Panc1*	1.0E-04
	hCD8^{+} T Cell IFN-γ relative MFI	Lenti-hId3+*nt*	Lenti-hId3+*Panc1*	3.8E-05
	hCD56^{+} NK Cell IFN-γ relative MFI	Lenti-hId3+*nt*	Lenti-hId3+*Panc1*	3.1E-05
Figure 6h	Nkp46^{+} NK cellsIFN-γ relative MFI	BMDM Lenti ctrl	BMDM Lenti-m*Id3*	2.1E-05
	CD8^{+} T cells IFN-γ relative MFI	BMDM Lenti ctrl	BMDM Lenti-m*Id3*	3.2E-05

Supplementary information Table1. Statistical P value for main figures.
Statistical P values for indicated figures are shown. p<0.0001.

Figures	Y title	Group1	Group2	Group2 vs Group1 p Value.
Extended Data Figure 1j	Liver Tim4^{+}cells / g (x10^{6})	d0	d0.5	7.6E-11
	Liver Tim4^{+}cells / g (x10^{6})	d0	d1	6.2E-11
	Liver Tim4^{+}cells / g (x10^{6})	d0	d7	2.4E-09
	Liver Tim4^{+}cells / g (x10^{6})	d0	d14	4.4E-06
Extended Data Figure 1m	Oncosphere size μm^{2} ×10^{3}	CD9^{+}CD133^{+}	CD9^{-}CD133^{-}	<1E-15
Extended Data Figure 2k	GFP^{+}(%) in *Cx3cr1*$^{gfp/+}$ mice	Tim4^{+}_no tumor	Tim4-MHCII^{+}_no tumor	5.1E-14
	GFP^{+}(%) in *Cx3cr1*$^{gfp/+}$ mice	Tim4^{+}_KPC-1	Tim4-MHCII^{+}_KPC-1	5.0E-14
	GFP^{+}(%) in *Cxcr4*$^{gfp/+}$ mice	Tim4^{+}_no tumor	Tim4-MHCII^{+}_no tumor	1.1E-05
	GFP^{+}(%) in *Cxcr4*$^{gfp/+}$ mice	Tim4^{+}_no tumor	Tim4-MHCII^{-}_no tumor	1.5E-05
	GFP^{+}(%) in *Cxcr4*$^{gfp/+}$ mice	Tim4^{+}_KPC-1	Tim4-MHCII^{+}_KPC-1	4.7E-08
	GFP^{+}(%) in *Cxcr4*$^{gfp/+}$ mice	Tim4^{+}_KPC-1	Tim4-MHCII^{-}_KPC-1	1.0E-05
	tdT^{+}(%) in *Cxcr4*^{CreERT2}*R26*$^{LSL-tdT}$ 4OH-TAM @6w	Tim4^{+}_no tumor	Tim4-MHCII^{+}_no tumor	2.8E-05
	tdT^{+}(%) in *Cxcr4*^{CreERT2}*R26*$^{LSL-tdT}$ 4OH-TAM @6w	Tim4^{+}_KPC-1	Tim4-MHCII^{+}_KPC-1	4.2E-10
	tdT^{+}(%) in *Cxcr4*^{CreERT2}*R26*$^{LSL-tdT}$ 4OH-TAM @6w	Tim4^{+}_KPC-1	Tim4-MHCII^{-}_KPC-1	3.1E-09
Extended Data Figure 3g	MFI CK19 in Tim4^{+} cells	Control	KPC #2	7.5E-13
	MFI CK19 in Tim4^{+} cells	Control	KPC #3	5.6E-12
Extended Data Figure 5a	CCL3 Relative MFI in KCs	Control	KPC	<1E-15
	CCL4 Relative MFI in KCs	Control	KPC	<1E-15
	CCL5 Relative MFI in KCs	Control	KPC	<1E-15
	IL12 Relative MFI in KCs	Control	KPC	<1E-15
	IL15 Relative MFI in KCs	Control	KPC	<1E-15
	IL18 Relative MFI in KCs	Control	KPC	<1E-15
Extended Data Figure 6a	SIRPA relative MFI in Tim4^{+}CD206^{hi} cells	Id3$^{f/f}$_no tumor	Clec4f^{Cre}Id3$^{f/f}$_no tumor	9.2E-06
	SIRPA relative MFI in Tim4^{+}CD206^{hi} cells	Id3$^{f/f}$_KPC-1	Clec4f^{Cre}Id3$^{f/f}$_KPC-1	6.8E-07
Extended Data Figure 6i	*Il18* relative mRNA	Id3$^{f/f}$+nt	Id3$^{f/f}$+KPC	3.8E-05
	Il18 relative mRNA	Id3$^{f/f}$+KPC	Clec4f^{Cre} Id3$^{f/f}$+KPC	8.3E-05
Extended Data Figure 6j	IFN-γ relative MFI	Id3$^{f/f}$+nt	Id3$^{f/f}$+KPC	8.1E-05
Extended Data Figure 7a	Dectin1 relative MFI	Clec4f^{Cre}Id3$^{f/f}$ + α-IgG	Clec4f^{Cre}Id3$^{f/f}$ + α-SIRPA	8.3E-06
	CCL3 relative MFI	Id3$^{f/f}$ + α-IgG_ 0-50μm	Clec4f^{Cre}Id3$^{f/f}$ + α-IgG_ 0-50μm	3.2E-14
	CCL3 relative MFI	Clec4f^{Cre}Id3$^{f/f}$ + α-IgG_ 0-50μm	Clec4f^{Cre}Id3$^{f/f}$ + α-SIRPA_ 0-50μm	4.8E-14
	CCL3 relative MFI	Id3$^{f/f}$ + α-IgG_core	Clec4f^{Cre}Id3$^{f/f}$ + α-IgG_core	4.6E-08
	CCL3 relative MFI	Clec4f^{Cre}Id3$^{f/f}$ + α-IgG_core	Clec4f^{Cre}Id3$^{f/f}$ + α-SIRPA_core	3.0E-08
	CCL4 relative MFI	Id3$^{f/f}$ + α-IgG_ 0-50μm	Clec4f^{Cre}Id3$^{f/f}$ + α-IgG_ 0-50μm	<1E-15
	CCL4 relative MFI	Clec4f^{Cre}Id3$^{f/f}$ + α-IgG_ 0-50μm	Clec4f^{Cre}Id3$^{f/f}$ + α-SIRPA_ 0-50μm	3.0E-14
	CCL4 relative MFI	Id3$^{f/f}$ + α-IgG_core	Clec4f^{Cre}Id3$^{f/f}$ + α-IgG_core	<1E-15
	CCL4 relative MFI	Clec4f^{Cre}Id3$^{f/f}$ + α-IgG_core	Clec4f^{Cre}Id3$^{f/f}$ + α-SIRPA_core	<1E-15
	CCL5 relative MFI	Id3$^{f/f}$ + α-IgG_ 0-50μm	Clec4f^{Cre}Id3$^{f/f}$ + α-IgG_ 0-50μm	<1E-15
	CCL5 relative MFI	Clec4f^{Cre}Id3$^{f/f}$ + α-IgG_ 0-50μm	Clec4f^{Cre}Id3$^{f/f}$ + α-SIRPA_ 0-50μm	<1E-15
	CCL5 relative MFI	Id3$^{f/f}$ + α-IgG_core	Clec4f^{Cre}Id3$^{f/f}$ + α-IgG_core	1.4E-11
	CCL5 relative MFI	Clec4f^{Cre}Id3$^{f/f}$ + α-IgG_core	Clec4f^{Cre}Id3$^{f/f}$ + α-SIRPA_core	1.2E-09
Extended Data Figure 7h	% Cells	KPC-Cd47$^{+/+}$-gfp	KPC-Cd47$^{-/-}$-tdT	5.0E-07
Extended Data Figure 8g	SIRPA relative MFI	WT _PBS	Id3 deficient_PBS	3.8E-08
	SIRPA relative MFI	Id3 deficient_PBS	Id3 deficient_LPS	6.7E-05
Extended Data Figure 8h	SIRPA relative MFI	Control_PBS	Control_LPS	2.7E-14
	SIRPA relative MFI	Control_LPS	Lenti-Id3_LPS	1.9E-14
	SIRPA relative MFI	Scramble_LPS	sh-E2A_LPS	1.9E-14
	SIRPA relative MFI	Scramble_LPS	sh-Elk1_LPS	1.9E-14
Extended Data Figure 9h	TIMD4 Expression level (PDAC.)	KC	TAMs	3.6E-86
	SIRPA Expression level (PDAC.)	KC	TAMs	1.5E-12
	IL18 Expression level (PDAC.)	KC	TAMs	3.7E-09
	TIMD4 Expression level (CRC.)	KC	TAMs	4.5E-12
	CCL3 Expression level (CRC.)	KC	TAMs	6.4E-15
	CCL4 Expression level (CRC.)	KC	TAMs	1.1E-26

Supplementary information Table2. Statistical P value for extended data figures.
Statistical P values for indicated figures are shown. $p<0.0001$.
