# Supplementary material for: The nuclear factor ID3 endows macrophages with a potent anti-tumour activity
Source: Nature. 2024 Feb 7;626(8000):864–73. doi: 10.1038/s41586-023-06950-4 (PMC10881399; doi:10.1038/s41586-023-06950-4)
Supplement: Supplementary file 2 — Reporting Summary [file 41586_2023_6950_MOESM2_ESM.pdf]

nature portfolio

Corresponding author(s): Frederic Geissmann

Last updated by author(s): 2023.11.14

Reporting Summary

Nature Portfolio wishes to improve the reproducibility of the work that we publish. This form provides structure for consistency and transparency in reporting. For further information on Nature Portfolio policies, see our Editorial Policies and the Editorial Policy Checklist.

Statistics

For all statistical analyses, confirm that the following items are present in the figure legend, table legend, main text, or Methods section.

n/a	Confirmed	
☐	☒	The exact sample size (*n*) for each experimental group/condition, given as a discrete number and unit of measurement
☐	☒	A statement on whether measurements were taken from distinct samples or whether the same sample was measured repeatedly
☐	☒	The statistical test(s) used AND whether they are one- or two-sided<br>*Only common tests should be described solely by name; describe more complex techniques in the Methods section.*
☒	☐	A description of all covariates tested
☐	☒	A description of any assumptions or corrections, such as tests of normality and adjustment for multiple comparisons
☐	☒	A full description of the statistical parameters including central tendency (e.g. means) or other basic estimates (e.g. regression coefficient) AND variation (e.g. standard deviation) or associated estimates of uncertainty (e.g. confidence intervals)
☐	☒	For null hypothesis testing, the test statistic (e.g. *F*, *t*, *r*) with confidence intervals, effect sizes, degrees of freedom and *P* value noted<br>*Give P values as exact values whenever suitable.*
☒	☐	For Bayesian analysis, information on the choice of priors and Markov chain Monte Carlo settings
☒	☐	For hierarchical and complex designs, identification of the appropriate level for tests and full reporting of outcomes
☒	☐	Estimates of effect sizes (e.g. Cohen's *d*, Pearson's *r*), indicating how they were calculated

Our web collection on statistics for biologists contains articles on many of the points above.

Software and code

Policy information about availability of computer code

Data collection	IVIS Spectrum for Bioluminescence imaging.<br>Leica DM IL inverted phase contrast microscope for Oncosphere imaging.<br>LSR Fortessa for Flow cytometry data.<br>GUAVA easyCyte HT for cell numbers.<br>Aria III BD cell sorter for cell sorting data.<br>Quant Studio 6 Flex System for Q_PCR data.<br>Axio Lab.A1 microscope for Cytospin data.<br>Zeiss LSM880 confocal microscope for Immunofluorescence ,wholemount imaging, time lapse imaging.<br>HiSeq 4000 for RNAseq data.
Data analysis	In vitro Oncosphere formation: ImageJ1.51.<br>Flow cytometry, cell sorting: FlowJo 10.6 .<br>Cell counting: GUAVA easyCyte HT.<br>Bioluminescence imaging: LivingImage 2.60.1.<br>Immunoflurescence imaging, time lapse imaging: Imaris9.3.1.<br>Single cell RNAseq:Seurat" package (4.3.0) ,R studio (4.2.0).<br>BulkRNAseq:STAR v2.7.10a.DESeq2 1.34.0<br>Identification of candidate functional E2A and ELK1 motifs:Python 3, Keras 2.3.1, tensorflow 2.1.0, scikit-learn 0.21.3, deeplift 0.6.10.0, biopython 1.76.

For manuscripts utilizing custom algorithms or software that are central to the research but not yet described in published literature, software must be made available to editors and reviewers. We strongly encourage code deposition in a community repository (e.g. GitHub). See the Nature Portfolio guidelines for submitting code & software for further information.

Data

Policy information about availability of data

All manuscripts must include a data availability statement. This statement should provide the following information, where applicable:

- Accession codes, unique identifiers, or web links for publicly available datasets
- A description of any restrictions on data availability
- For clinical datasets or third party data, please ensure that the statement adheres to our policy

Public data used in this study, Single-cell RNA-seq analysis: The CRC dataset:GSE146409, The PDAC dataset : GSE205013, the nontumor control dataset : GSE115469. Identification of candidate functional E2A and ELK1 motifs: GSE128338.mouse reference genome GRCm39.human protein atlas (https://www.proteinatlas.org/). All the codes, analyzed RDS files, and the original data sets are available via the following link (https://doi.org/10.5281/zenodo.10121153).Bulk RNAseq raw data are deposited to the GEO repository with GEO accession number: GSE234638

Research involving human participants, their data, or biological material

Policy information about studies with human participants or human data. See also policy information about sex, gender (identity/presentation), and sexual orientation and race, ethnicity and racism.

Reporting on sex and gender	sex was not considered in this study
Reporting on race, ethnicity, or other socially relevant groupings	The study did not collect any information about race, ethnicity information
Population characteristics	Patient1: Age62/M,cTxNxM1 (Stage IV). Treatment: Taxoprexin (4 cycles); PD; Gemcitabine (1 cycle). Mutation: Kras, p.G12D; TP53, p.T155P. Patient2: Age57/M, cT3NxM1 (Stage IV). Treatment: Taxoprexin (1 cycle); PD; Gemcitabine (4 cycles). Mutation: Kras, p.Q61H; TP53: p.L257P. Patient3: Age60/M, no treatment. Mutation: Kras, p.G12D; TP53, p.L344P.
Recruitment	Autopsy from diagnosed PDAC patients.
Ethics oversight	Human tissues were obtained with patient-informed consent and used under approval by the Institutional Review Boards from Memorial Sloan Kettering Cancer Center(IRB protocols #15-021).

Note that full information on the approval of the study protocol must also be provided in the manuscript.

Field-specific reporting

Please select the one below that is the best fit for your research. If you are not sure, read the appropriate sections before making your selection.

☒ Life sciences ☐ Behavioural & social sciences ☐ Ecological, evolutionary & environmental sciences

For a reference copy of the document with all sections, see nature.com/documents/nr-reporting-summary-flat.pdf

Life sciences study design

All studies must disclose on these points even when the disclosure is negative.

Sample size	No statistical methods were used to predetermine sample size, Sample size for each experiment is indicated in the figures and figure legends. Sample sizes were estimated based on preliminary experiments, with an effort to achieve a minimum of n=3, mostly n=20 mice per treatment group, which proved sufficient to determine reproducible results.
Data exclusions	No data was excluded from the analysis presented in this study.
Replication	All experiments were repeated multiple times with similar results. Replicates were stated in the Figure Legends and methods/Statistical analysis.Key Observations from RNA-seq studies have been validated with Flow cytometry and RT_qPCR.
Randomization	For in vivo experiments, mice were randomly assigned to different groups.
Blinding	For in-vivo study, blinding was performed during tumor cells injection and tumor burden measurements, For Bulk RNAseq, time lapse imaging, blinding was performed from sample collection to data analysis. For other experiments, investigators were not blind to group allocation as this information was essential for experiment conducting.

Reporting for specific materials, systems and methods

We require information from authors about some types of materials, experimental systems and methods used in many studies. Here, indicate whether each material, system or method listed is relevant to your study. If you are not sure if a list item applies to your research, read the appropriate section before selecting a response.

Materials & experimental systems

n/a	Involved in the study
☐	☒ Antibodies
☐	☒ Eukaryotic cell lines
☒	☐ Palaeontology and archaeology
☐	☒ Animals and other organisms
☒	☐ Clinical data
☒	☐ Dual use research of concern
☒	☐ Plants

Methods

n/a	Involved in the study
☐	☒ ChIP-seq
☐	☒ Flow cytometry
☒	☐ MRI-based neuroimaging

Antibodies

Antibodies used

Antibodies are listed in Supplementary table 5.The following antibodies were used in immunofluorescence assay.
Primary antibodies:
anti-mouse-F4/80-eF450/AF647/AF488/eF570(1:200, BM8, eBioscience),
anti-mouse Tim4-AF647/PE(1:200, RMT4-54,Biolegend),
anti-mouse CD45.1-AF488(1:200, A20, Biolegend),
Chicken-anti-GFP (1:500, A10262,Invitrogen, recognize YFP),
Rabbit-anti-RFP (1:200, 600-401-379, Rockland),
Goat-anti mouse Clec4f (1:200, AF2784,R&D system),
anti-mouse CCL3(1:200, 50-7532-82,Thermo Fisher Scientific),
anti-mouse CCL4(1:200, AF-451-NA,Thermo Fisher Scientific),
anti-mouse CCL5(1:200, 701030,Thermo Fisher Scientific),
anti-mouse IL12p70(1:200, MM121B,Thermo Fisher Scientific),
anti-mouse IL15(1:200, AF447-SP, Thermo Fisher Scientific),
anti-mouse IL18(1:200, PA5-79481,Thermo Fisher Scientific) antibodies,
anti-human CD14-AF488(1:200, 561706, BD),
sheep-anti-human CK19(1:200, AF3506, R&D system),
rabbit-anti-human Tim4(1:200, PA5-53346, Thermo Fisher Scientific),
Goat-anti-human IL12(1:200, AF-219-NA, R&D system),
Goat-anti-human IL18(1:200, AF2548, R&D system),
Mouse-anti-human IL15(1:200, MAB2471, R&D system),
Goat-anti-human CCL3(1:200, AF-270-NA, R&D system),
Goat-anti-human CCL4(1:200, AF-271-NA, R&D system),
Goat-anti-human CCL5(1:200, AF-278-NA, R&D system).
Secondary antibodies:
anti-chicken Alexa Fluor 488 (1:500; a11039,Thermo Fisher Scientific),
anti-rabbit Alexa Fluor 555 (1:500, A32794 ,Thermo Fisher Scientific),
anti-rabbit Alexa Fluor 647(1:500, A32795, Thermo Fisher Scientific),
anti-goat Alexa Fluor 555 (1:500�a32816, Thermo Fisher Scientific),
anti-goat Alexa Fluor 647(1:500�a21447, Thermo Fisher Scientific),
anti-goat Alexa Fluor 488 (1:500�a32814,Thermo Fisher Scientific),
anti-sheep Alexa Fluor 568(1:500, A21099, Thermo Fisher Scientific),
Streptavidin Alexa Fluor 647 (1:500�405237, BioLegend).
anti-sheep Alexa Fluor 568(1:500, A21099, Thermo Fisher Scientific),
anti-mouse Alexa Fluor 555(1:500, A-31570, Thermo Fisher Scientific).
The following antibodies were used in Flow cytometry assay:
anti-mouse-CD45-APC-eF780(1:200, 30-F11,ebioscience),
anti-mouse-CD45.1-AF488/PE (1:200, A20,Biolegend),
anti-mouse-CD45.2-APC-cy7(1:200, 104,Biolegend),
anti-mouse-Tim4-AF647/PE(1:200, RMT4-54,Biolegend),
anti-mouse-CD11b-PE-Cy7(1:200, M1/70,BD Biosciences),
anti-mouse-F4/80-eF450/BV605(1:200, BM8,ebioscience),
anti-mouse-Gr1-BV711(1:200, RB6-8C5,BD Biosciences),
anti-mouse-I-A/I-E-AF700(1:200, M5/114.15.2,Biolegend),
anti-mouse-CD11c-BV605(1:200, N418,Biolegend),
anti-mouse-Ly6C-BV510/BV786/AF488(1:200, HK1.4,Biolegend),
anti-mouse-CD117 (c-KIT)-BV605(1:200, 2B8,BD Biosciences)
anti-mouse-CD64-BV711/APC(1:200, X54-5/7.1,Biolegend)
anti-mouse-CD115-BV605/APC(1:200, AFS98,Biolegend)
anti-mouse-CD19-BV711 (1:200, 1D3,Biolegend)
anti-mouse-CD19-BV711 (1:200, 1D3/CD19,Biolegend)
anti-mouse-CD3-BV711/BV421(1:200, 145-2C11,Biolegend)
anti-mouse-CD3-APC-eF780 (1:200,145-2C11,ebioscience)
anti-mouse-CD335-AF647/BV711/percp-cy5.5 (1:200,29A1.4,Biolegend)
anti-mouse-CD48-APC(1:200,HM48-1,Biolegend)
anti-mouse-CD150 (SLAM)-PE-Cy7(1:200,TC15-12F12.2,Biolegend)

anti-mouse-Siglec F-AF647/BV711(1:200,E50-2440,BD Biosciences)
anti-mouse-Ly6G-BV421/PE/Dazzle594(1:200,1A8,Biolegend)
anti-mouse-Sca1-BV421(1:200,D7,BD Biosciences)
anti-mouse-CD64-BV711(1:200,X54-5/7.1,Biolegend)
anti-mouse-CD16/32 (1:100, 93,Biolegend)
anti-mouse-CD47-FITC/PE/AF647(1:200,miap301,Biolegend)
anti-mouse-CD133-PE-Cy7(1:200,315-2C11,Biolegend)
anti-mouse-CD44-BV785(1:200,IM7,Biolegend)
anti-mouse-CD9-AF700(1:200,KMC 8,ebioscience)
anti-mouse-CD24-BV605(1:200,M1/69,Biolegend)
anti-mouse-Epcam-BV711(1:200,G8.8,Biolegend)
anti-mouse-Sirpa-AF488/PE/PE-Cy7 (1:200,P84,Biolegend)
anti-mouse-CD206-BV785(1:200,C068C2,Biolegend)
anti-mouse-Clec7a-PE/APC(1:200,RH1,Biolegend)
anti-mouse-CD1d PBS-57 tetrame-AF647(1:200,NIH Tetramer Core Facility)
anti-mouse-TCRβ-PE-Cy7(1:200,H57-597,BD Biosciences)
anti-mouse-TCRγδ-BV786(1:200,GL3,BD Biosciences)
anti-mouse-CD4-BV421(1:200,RM4-5,Biolegend)
anti-mouse-CD8-BV605/AF647(1:200,53-6.7,Biolegend)
anti-mouse-IFNg-PE-Cy7(1:200,XMG1.2,Biolegend)
anti-mouse-TNFa-AF647(1:200,MP6-XT22,Biolegend)
anti-human-CD8-PE(1:200,SK1,Biolegend)
anti-human-CD56-PE(1:200,HCD56,Biolegend)
anti-human-IFNg-APC(1:200,B27,BD Biosciences)
anti-human-TNFa-BV650(1:200,MAb11,Biolegend)

Validation

all antibodies used are commercially available and catalog numbers/clones are stated in Supplementary table 5, detailed as follow:
anti-mouse-CD45(30-F11,ebioscience) https://www.thermofisher.com/antibody/product/CD45-Antibody-clone-30-F11-Monoclonal/47-0451-82?gclid=CjwKCAiA0syqBhBxEiwAeNx9N0Wh3nljQ1xtqQcJSwawK0O6o3sfnM6ldsEKIcKH4aP4Jn5iVIWxEBoC1ZUQAvD_BwE&ef_id=CjwKCAiA0syqBhBxEiwAeNx9N0Wh3nljQ1xtqQcJSwawK0O6o3sfnM6ldsEKIcKH4aP4Jn5iVIWxEBoC1ZUQAvD_BwE:G:s&s_kwcid=AL!3652!3!278870232429!!!g!!!1454324556!63404918784&cid=bid_pca_frg_r01_co_cp1359_pjt0000_bid00000_0se_gaw_dy_pur_con
anti-mouse-CD45.1(A20,Biolegend) https://www.biolegend.com/en-us/products/alexa-fluor-488-anti-mouse-cd45-1-antibody-3103
anti-mouse-CD45.2(104,Biolegend) https://www.biolegend.com/en-us/products/apc-cyanine7-anti-mouse-cd45-2-antibody-3906
anti-mouse-Tim4(RMT4-54,Biolegend) https://www.biolegend.com/en-us/products/alexa-fluor-647-anti-mouse-tim-4-antibody-5243
anti-mouse-CD11b(M1/70,BD Biosciences) https://www.bdbiosciences.com/en-us/products/reagents/flow-cytometry-reagents/research-reagents/single-color-antibodies-ruo/pe-cy-7-rat-anti-cd11b.552850
anti-mouse-F4/80(BM8,ebioscience) https://www.thermofisher.com/antibody/product/F4-80-Antibody-clone-BM8-Monoclonal/48-4801-82
anti-mouse-Gr1(RB6-8C5,BD Biosciences) https://www.bdbiosciences.com/en-us/products/reagents/flow-cytometry-reagents/research-reagents/single-color-antibodies-ruo/bv711-rat-anti-mouse-ly-6g-and-ly-6c.740658
anti-mouse-I-A/I-E(M5/114.15.2,Biolegend) https://www.biolegend.com/en-us/products/alexa-fluor-700-anti-mouse-i-a-i-e-antibody-3413
anti-mouse-CD11c(N418,Biolegend) https://www.biolegend.com/en-us/products/brilliant-violet-605-anti-mouse-cd11c-antibody-7865
anti-mouse-Ly6C(HK1.4,Biolegend) https://www.biolegend.com/en-us/products/brilliant-violet-785-anti-mouse-ly-6c-antibody-11982
anti-mouse-CD117 (c-KIT)(2B8,BD Biosciences) https://www.biolegend.com/en-us/products/brilliant-violet-605-anti-mouse-cd117-c-kit-antibody-16969
anti-mouse-CD64(X54-5/7.1,Biolegend) https://www.biolegend.com/en-us/products/brilliant-violet-711-anti-mouse-cd64-fcgammari-antibody-9920
anti-mouse-CD115(AFS98,Biolegend) https://www.biolegend.com/en-us/products/brilliant-violet-605-anti-mouse-cd115-csf-1r-antibody-9013
anti-mouse-CD19(1D3,Biolegend) https://www.bdbiosciences.com/en-us/products/reagents/flow-cytometry-reagents/research-reagents/single-color-antibodies-ruo/bv711-rat-anti-mouse-cd19.563157
anti-mouse-CD19(1D3/CD19,Biolegend) https://www.biolegend.com/en-us/products/alexa-fluor-700-anti-mouse-cd19-antibody-22036
anti-mouse-CD3(145-2C11,Biolegend) https://www.biolegend.com/en-us/products/brilliant-violet-711-anti-mouse-cd3epsilon-antibody-11975
anti-mouse-CD3(145-2C11,ebioscience) https://www.thermofisher.com/antibody/product/CD3e-Antibody-clone-145-2C11-Monoclonal/47-0031-82
anti-mouse-CD335 (NKp46,Biolegend) https://www.bdbiosciences.com/en-us/products/reagents/flow-cytometry-reagents/research-reagents/single-color-antibodies-ruo/bv711-rat-anti-mouse-cd335-nkp46.740822
anti-mouse-CD48(29A1.4,Biolegend) https://www.biolegend.com/en-us/products/apc-anti-mouse-cd48-antibody-3622
anti-mouse-CD150 (SLAM)(TC15-12F12.2,Biolegend) https://www.biolegend.com/en-us/products/pe-cyanine7-anti-mouse-cd150-slam-antibody-3056
anti-mouse-Siglec F(E50-2440,BD Biosciences) https://www.bdbiosciences.com/en-us/products/reagents/flow-cytometry-reagents/research-reagents/single-color-antibodies-ruo/alexa-fluor-647-rat-anti-mouse-siglec-f.562680
anti-mouse-Ly6G(1A8,Biolegend) https://www.biolegend.com/en-us/products/pe-dazzle-594-anti-mouse-ly-6g-antibody-12246
anti-mouse-Sca1(D7,BD Biosciences) https://www.bdbiosciences.com/en-us/products/reagents/flow-cytometry-reagents/research-reagents/single-color-antibodies-ruo/bv421-rat-anti-mouse-ly-6a-e.562729
anti-mouse-CD64(X54-5/7.1,Biolegend) https://www.biolegend.com/en-us/products/brilliant-violet-711-anti-mouse-cd64-fcgammari-antibody-9920
anti-mouse-CD16/32 (93,Biolegend) https://www.biolegend.com/en-us/products/purified-anti-mouse-cd16-32-antibody-190
anti-mouse-CD47(miap301,Biolegend) https://www.biolegend.com/en-us/products/pe-anti-mouse-cd47-antibody-4926
anti-mouse-CD133(315-2C11,Biolegend) https://www.biolegend.com/en-us/products/pe-cyanine7-anti-mouse-cd133-antibody-10193

anti-mouse-CD44(IM7,Biolegend) https://www.biolegend.com/en-us/products/brilliant-violet-785-anti-mouse-human-cd44-antibody-7959
anti-mouse-CD9(KMC 8,ebioscience) https://www.thermofisher.com/antibody/product/CD9-Antibody-clone-eBioKMC8-KMC8-Monoclonal/56-0091-82
anti-mouse-CD24(M1/69,Biolegend) https://www.biolegend.com/en-us/products/brilliant-violet-605-anti-mouse-cd24-antibody-9691
anti-mouse-Epcam(G8.8,Biolegend) https://www.biolegend.com/en-us/products/brilliant-violet-711-anti-mouse-cd326-ep-cam-antibody-13763
anti-mouse-Sirpa(P84,Biolegend) https://www.biolegend.com/en-us/products/alexa-fluor-488-anti-mouse-cd172a-sirpalpha-antibody-14089
anti-mouse-CD206(C068C2,Biolegend) https://www.biolegend.com/en-us/products/brilliant-violet-785-anti-mouse-cd206-mmr-antibody-12013
anti-mouse-Clec7a(RH1,Biolegend) https://www.biolegend.com/en-us/products/pe-anti-mouse-cd369-dectin-1-clec7a-antibody-8102
anti-mouse-TCRβ(H57-597,BD Biosciences) https://www.bdbiosciences.com/en-us/products/reagents/flow-cytometry-reagents/research-reagents/single-color-antibodies-ruo/pe-cy-7-hamster-anti-mouse-tcr-chain.560729
anti-mouse-TCRγδ(GL3,BD Biosciences) https://www.bdbiosciences.com/en-us/products/reagents/flow-cytometry-reagents/research-reagents/single-color-antibodies-ruo/bv786-hamster-anti-mouse-t-cell-receptor.744117
anti-mouse-CD4(RM4-5,Biolegend) https://www.biolegend.com/en-us/products/brilliant-violet-421-anti-mouse-cd4-antibody-7349
anti-mouse-CD8(53-6.7,Biolegend) https://www.biolegend.com/en-us/products/brilliant-violet-605-anti-mouse-cd8a-antibody-7636
anti-mouse-IFNg(XMG1.2,Biolegend) https://www.biolegend.com/en-us/products/pe-cyanine7-anti-mouse-ifn-gamma-antibody-5865
anti-mouse-TNFa(MP6-XT22,Biolegend) https://www.biolegend.com/en-us/products/alexa-fluor-647-anti-mouse-tnf-alpha-antibody-2724
anti-human-CD8(SK1,Biolegend) https://www.biolegend.com/en-us/products/pe-anti-human-cd8-antibody-6247
anti-human-CD56(HCD56,Biolegend) https://www.biolegend.com/en-us/products/pe-anti-human-cd56-ncam-antibody-3796
anti-human-IFNg(B27,BD Biosciences) https://www.bdbiosciences.com/en-us/products/reagents/flow-cytometry-reagents/research-reagents/single-color-antibodies-ruo/apc-mouse-anti-human-ifn.562017
anti-human-TNFa(MAb11,Biolegend) https://www.biolegend.com/en-us/products/brilliant-violet-650-anti-human-tnf-alpha-antibody-7680
Rabbit-anti-mouse-Lamb1(ab24170,Abcam) https://www.abcam.com/products/primary-antibodies/lamp1-antibody-lysosome-marker-ab24170.html
Rat-anti-mouse-Lamp1(1D4B,Biolegend) https://www.biolegend.com/en-us/products/alexa-fluor-647-anti-mouse-cd107a-lamp-1-antibody-3589
Goat-anti-mouse-Clec4f(AF2784,R&D system) https://www.rndsystems.com/products/mouse-clec4f-clecsf13-antibody_af2784?gclid=CjwKCAiA0syqBhBxEiwAeNx9NzuVAyZtHcQ2t2mAX-HxQZON-V4an6sh-7454lXYNRAJ5N6veglKKxoCHHsQAvD_BwE&gclsrc=aw.ds
Rabbit-anti-mouse-Elk1(9182S,Cell signaling technology) https://www.cellsignal.com/products/primary-antibodies/elk-1-antibody/9182
Rat-anti-mouse-CCL3(50-7532-82,Thermo Fisher Scientific) https://www.thermofisher.com/antibody/product/CCL3-MIP-1-alpha-Antibody-clone-DNT3CC-Monoclonal/50-7532-82
Goat-anti-mouse-CCL4(AF-451-NA,R&D system) https://www.rndsystems.com/products/mouse-ccl4-mip-1beta-antibody_af-451-na?gclid=CjwKCAiA0syqBhBxEiwAeNx9N83iPzTpnzHXEO1KGjN-zOqjiuMOP4ewN1ty2NgN0GAz7s691FOeLxoC8TAQAvD_BwE&gclsrc=aw.ds
Rabbit-anti-mouse-CCL5(701030,Thermo Fisher Scientific) https://www.thermofisher.com/antibody/product/CCL5-RANTES-Antibody-clone-25H14L17-Recombinant-Monoclonal/701030
Rat-anti-mouse-IL12p70(MM121B,Thermo Fisher Scientific) https://www.thermofisher.com/antibody/product/IL-12-p70-Antibody-clone-C17-8-Monoclonal/MM121B
Goat-anti-mouse-IL15(AF447-SP,R&D system) https://www.rndsystems.com/products/mouse-il-15-antibody_af447
Rabbit-anti-mouse-IL18(PA5-79481) https://www.thermofisher.com/antibody/product/IL-18-Antibody-Polyclonal/PA5-79481
Mouse-anti-human CD14(M5E2,BD Biosciences) https://www.bdbiosciences.com/en-us/products/reagents/flow-cytometry-reagents/research-reagents/single-color-antibodies-ruo/alexa-fluor-488-mouse-anti-human-cd14.561706
Rabbit-anti-human TIMD4(PA5-53346,Thermo Fisher Scientific) https://www.thermofisher.com/antibody/product/TIMD4-Antibody-Polyclonal/PA5-53346
Sheep-anti-human CK19(AF3506,R&D system) https://www.rndsystems.com/products/human-cytokeratin-19-antibody_af3506
Mouse-anti-human CK19(ab7754,Abcam) https://www.abcam.com/products?keywords=Cytokeratin+19&gad=1&gclid=CjwKCAiA0syqBhBxEiwAeNx9N3BdLDzd2ZIGgf3XWLlxor2FvGnraTGiBLFpLZzOdH6LyL-Ky5C8URoCPIQQAvD_BwE&gclsrc=aw.ds
Goat-anti-human IL12(AF-219-NA,R&D system) https://www.rndsystems.com/products/human-il-12-antibody_af-219-na
Goat-anti-human IL18(AF2548,R&D system) https://www.rndsystems.com/products/human-rhesus-macaque-il-18-il-1f4-antibody_af2548
Mouse-anti-human IL15(MAB2471,R&D system) https://www.rndsystems.com/products/human-il-15-antibody-34559_mab2471
Goat-anti-human CCL3(AF-270-NA,R&D system) https://www.rndsystems.com/products/human-ccl3-mip-1alpha-antibody_af-270-na
Goat-anti-human CCL4(AF-271-NA,R&D system) https://www.rndsystems.com/products/human-ccl4-mip-1beta-antibody_af-271-na
Goat-anti-human CCL5(AF-278-NA,R&D system) https://www.rndsystems.com/products/human-ccl5-rantes-antibody_af-278-na
Goat anti-rabbit IgG(a11034,Thermo Fisher Scientific) https://www.thermofisher.com/antibody/product/Goat-anti-Rabbit-IgG-H-L-Highly-Cross-Adsorbed-Secondary-Antibody-Polyclonal/A-11034
Goat anti-rabbit IgG(a32795,Thermo Fisher Scientific) https://www.thermofisher.com/antibody/product/Donkey-anti-Rabbit-IgG-H-L-Highly-Cross-Adsorbed-Secondary-Antibody-Polyclonal/A32795
Goat anti-rabbit IgG(a21430,Thermo Fisher Scientific) https://www.thermofisher.com/antibody/product/Goat-anti-Rabbit-IgG-H-L-Cross-Adsorbed-Secondary-Antibody-Polyclonal/A-21430
Donkey anti-goat IgG(a32816,Thermo Fisher Scientific) https://www.thermofisher.com/antibody/product/Donkey-anti-Goat-IgG-H-L-Highly-Cross-Adsorbed-Secondary-Antibody-Polyclonal/A32816
Donkey anti-goat IgG(a32814,Thermo Fisher Scientific) https://www.thermofisher.com/antibody/product/Donkey-anti-Goat-IgG-H-L-Highly-Cross-Adsorbed-Secondary-Antibody-Polyclonal/A32814
Donkey anti-goat IgG(a21447,Thermo Fisher Scientific) https://www.thermofisher.com/antibody/product/Donkey-anti-Goat-IgG-H-L-Cross-Adsorbed-Secondary-Antibody-Polyclonal/A-21447

Goat-anti-Chicken IgG(a11039,Thermo Fisher Scientific) https://www.thermofisher.com/antibody/product/Goat-anti-Chicken-IgY-H-L-Secondary-Antibody-Polyclonal/A-11039
Donkey anti-Sheep IgG(A21099,Thermo Fisher Scientific) https://www.thermofisher.com/antibody/product/Donkey-anti-Sheep-IgG-H-L-Cross-Adsorbed-Secondary-Antibody-Polyclonal/A-21099
Donkey anti-Rabbit IgG (A32795,Thermo Fisher Scientific) https://www.thermofisher.com/antibody/product/Donkey-anti-Rabbit-IgG-H-L-Highly-Cross-Adsorbed-Secondary-Antibody-Polyclonal/A32795
Donkey anti-Rabbit IgG (A32794,Thermo Fisher Scientific) https://www.thermofisher.com/antibody/product/Donkey-anti-Rabbit-IgG-H-L-Highly-Cross-Adsorbed-Secondary-Antibody-Polyclonal/A32794
Donkey anti-Mouse IgG(A32766TR,Thermo Fisher Scientific) https://www.thermofisher.com/antibody/product/Donkey-anti-Mouse-IgG-H-L-Highly-Cross-Adsorbed-Secondary-Antibody-Polyclonal/A32766TR
Donkey anti-Mouse IgG(A-31570,Thermo Fisher Scientific) https://www.thermofisher.com/antibody/product/Donkey-anti-Mouse-IgG-H-L-Highly-Cross-Adsorbed-Secondary-Antibody-Polyclonal/A-31570
Streptavidin(405237,BioLegend) https://www.biolegend.com/en-us/products/alexa-fluor-647-streptavidin-9305
Chicken-anti-GFP(A10262,Thermo Fisher Scientific) https://www.thermofisher.com/antibody/product/GFP-Antibody-Polyclonal/A10262
Rabbit-anti-RFP(600-401-379,Rockland) https://www.rockland.com/categories/primary-antibodies/rfp-antibody-pre-adsorbed-600-401-379/?gad=1&gclid=CjwKCAiA0syqBhBxEiwAeNx9N5tsuMVFbCe23BzyQPUmvdS86suX3gpnUnuEkHqWfhXC07qhZyY_RRoC_fMQAvD_BwE
anti-mouse Dectin1(blockage)(R1-8g7,InvivoGen) https://www.invivogen.com/anti-mdectin1
anti-mouse-NK1.1(blockage)(BE0036,BioXcell) https://bioxcell.com/invivomab-anti-mouse-nk1-1-be0036?gad=1&gclid=CjwKCAiA0syqBhBxEiwAeNx9Nxt8Qgd8eOvuEcllXGCPClfYhuElX_wLypwqhBWJP5c7r1yn7QPJPBoCtVgQAvD_BwE
anti-mouse-CD8a(blockage)(BP0061,BioXcell) https://bioxcell.com/invivoplus-anti-mouse-cd8-alpha-bp0061
Control IgG(HRPN,BioXcell) https://bioxcell.com/invivomab-rat-igg1-isotype-control-anti-horseradish-peroxidase-be0088
Sirpa(P84,BioXcell) https://bioxcell.com/invivomab-anti-mouse-cd172a-sirp-alpha-be0322

Eukaryotic cell lines

Policy information about cell lines and Sex and Gender in Research

Cell line source(s)	Mouse cell lines: KPC-1, KPC-2,PAN02,MC38,B16F10,LLC1.Human cell lines: PANC-1. KPC-1,2 cancer cell lines derived from KPC pancreatic tumors by passaging in cell culture.
Authentication	Morphology , antibodies stain and PCR validate cell lines.
Mycoplasma contamination	No contamination
Commonly misidentified lines (See ICLAC register)	No commonly misidentified lines.

Animals and other research organisms

Policy information about studies involving animals; ARRIVE guidelines recommended for reporting animal research, and Sex and Gender in Research

Laboratory animals	Materials and Methods/Mice.Rosa26LSL-YFP , Rosa26LSL-tdT , Flt3Cre , Id3f/f , Id3-/- , Cx3cr1gfp/+ , CCR2-/- , Csf1rf/f , Spi1f/f , Cxcr4 gfp/+ , Cxcr4CreERT2 , Clec4fCre-tdT , Rosa26LSL-DTR , p48Cre , p53LSL-R172H , KrasLSL-G12D,CD45.1,C57BL/6J,Mice were housed in groups of no more than 5 per cage in standard closed plastic cages containing bedding, food and water. The room is controlled at stable temperature ~21-22°C with 30-70% humidity, 12light/12dark cycle(on from 6:00-18:00), 10-15 fresh air exchanges hourly.
Wild animals	No wild animals were employed in the study.
Reporting on sex	Sex was not considered in this study.
Field-collected samples	No field-collected samples were used in this study.
Ethics oversight	Materials and Methods/Mice. Animal procedures were performed in adherence with the Institutional Review Board (IACUC 15-04-006) at Memorial Sloan Kettering Cancer Center (MSKCC).

Note that full information on the approval of the study protocol must also be provided in the manuscript.

Plants

Seed stocks | N.A.

Novel plant genotypes | N.A.

Authentication | N.A.

ChIP-seq

Data deposition

☒ Confirm that both raw and final processed data have been deposited in a public database such as GEO.

☒ Confirm that you have deposited or provided access to graph files (e.g. BED files) for the called peaks.

Data access links *May remain private before publication.*	CHIPseq data are shown in Extended data Fig8c,d
Files in database submission	Available in GEO:GSE128338. ref.PMID: 32362324.
Genome browser session (e.g. UCSC)	UCSC

Methodology

Replicates	Materials and Methods/Identification of candidate functional E2A and ELK1 motifs.
Sequencing depth	libraries were size selected 250-500 bp using gel extraction using 10% TBE acrylamide gels. Libraries were single-end sequenced using either a HiSeq 4000 or a NextSeq 500 to a depth of 10-20 million reads.
Antibodies	Antibodies are shown in ref.PMID: 32362324.
Peak calling parameters	Peak files were merged with HOMER's mergePeaks and annotated with raw tag counts with HOMER's annotatePeaks using parameters -noadj, -size given. To annotate H3K27ac signal around ATAC-seq peaks the parameter -size 2000 was used.
Data quality	DESeq2 was used to identify the differentially bound H3K27ac signal or chromatin accessibility with FC > 2 and p-adj < 0.05. D
Software	Materials and Methods/Identification of candidate functional E2A and ELK1 motifs.

Flow Cytometry

Plots

Confirm that:

☒ The axis labels state the marker and fluorochrome used (e.g. CD4-FITC).

☒ The axis scales are clearly visible. Include numbers along axes only for bottom left plot of group (a 'group' is an analysis of identical markers).

☒ All plots are contour plots with outliers or pseudocolor plots.

☒ A numerical value for number of cells or percentage (with statistics) is provided.

Methodology

Sample preparation	Materials and Methods/Flow cytometry, cell sorting, cell counting.
Instrument	Flow cytometry was performed using a BD Biosciences LSRFortessa flow cytometer with Diva software.
Software	All data were analyzed using FlowJo 10.6 (Tree Star Inc.).
Cell population abundance	The purity of sorted samples was analyzed by flow cytometry on the same instruments used for sorting.

Gating strategy	Materials and Methods/Flow cytometry, cell sorting, cell counting/Gating strategy. Extended Data Fig1e, Extended Data Fig2j, Supplementary information Figure 1, 2, 3.

☒ Tick this box to confirm that a figure exemplifying the gating strategy is provided in the Supplementary Information.
